# Assessment of Feasibility and Patency of below the Knee Atherectomy Using the 1.5 mm Phoenix Catheter—A Retrospective Study

**DOI:** 10.3390/medicina58111594

**Published:** 2022-11-03

**Authors:** Arun Kumarasamy, Alexander Gombert, Julia Krabbe, Oliver Ruprecht, Michael J. Jacobs, Hanif Krabbe

**Affiliations:** 1 European Vascular Centre Aachen-Maastricht, Department of Vascular Surgery, Medical Faculty, University Hospital RWTH Aachen, Pauwelsstraße 30, 52074 Aachen, Germany; 2 Interventional Radiology, Krankenhaus Sachsenhausen, Schulstrasse 31, 60594 Frankfurt am Main, Germany; 3 Institute of Occupational, Social and Environmental Medicine, Medical Faculty, RWTH Aachen University, Pauwelsstraße 30, 52074 Aachen, Germany

**Keywords:** peripheral arterial disease, below the knee atherectomy

## Abstract

*Background and Objectives*: Peripheral arterial disease (PAD) contains a significant proportion of patients whose main pathology is located in the infragenicular arteries. The treatment of these patients requires a deliberate consideration due to the threat of possible complications of an intervention. In this retrospective study, the feasibility of a below-the-knee atherectomy (BTKA) via a 1.5 mm Phoenix atherectomy catheter and the patient outcome over the course of 6 months are investigated. *Materials and Methods*: The data of patients suffering from PAD with an infragenicular pathology treated via 1.5 mm Phoenix™ atherectomy catheter between March 2021 and February 2022 were retrospectively analyzed. Prior to the intervention, after 2 weeks and 6 months, the PAD stages were graded and ankle-brachial-indeces (ABI) were measured. *Results*: The study shows a significant improvement of ABI, both after 2 weeks and 6 months. Additionally, the number of PAD stage IV patients decreased by 15.2% over the course of 6 months, and 18.2% of the patients improved to PAD stage IIa. Only one bleeding complication on the puncture side occurred over the whole study, and no other complications were observed. *Conclusions*: Phoenix™ atherectomy usage in the BTKA area seems to be feasible and related to a favorable outcome in this retrospective study.

## 1. Introduction

Peripheral arterial disease (PAD), with its prevalence of 5.9% in the population aged ≥40 years, contains a significant proportion of patients whose main pathology is located in the infragenicular arteries; these changes are causal for up to 19.1% of PAD patients [1,2]. Treatment of these patients, like in any disease, should be based on a risk-benefits analysis [3]. In a chronic limb-threatening ischemia (CLTI), this equation seems to be significantly pushed towards the choice for treatment due to the severe physical constraint and poor outcome of the disease [4]. The choice of treatment gets more difficult if the symptoms according to the Rutherford/Fontaine classifications are moderate. In a compensated arterial system, treatment of ischemia might need deliberate consideration due to the threat of possible complications of an intervention. This issue may occur regardless of the treatment of choice: either angioplasty or artherectomy [5]. What seems to be a successful intervention at first can still lead to complications during follow-up. Studies show amputation rates of 10% and, additionally, the necessity for an open bypass surgery in 10% after different peripheral arterial interventions [6]. Still, atherectomy devices constitute a growing market with a rising costumer demand. Actually, it is considered as an add-on to the current portfolio of cardiovascular or radiologic clinics [7]. 

To the best of our knowledge, this study is the first to show results on a below-the-knee-focused atherectomy device with target vessels distal of the popliteal artery. This study mainly aims to evaluate the feasibility and safety of using such devices. To investigate these hypotheses, this study focuses on two questions: (1) Can a below-the-knee atherectomy (BTKA) be successfully performed in a routine setting with an appropriate complication rate? (2) Is there a relevant clinical benefit of BTKA application in patients suffering from different stages of PAD? Accordingly, a retrospective analysis of atherectomy procedures in a specialized center for peripheral arterial intervention was conducted.

## 2. Materials and Methods

All data collection including follow-up data patient inclusion criteria as well as statistical analysis, described according to The Strengthening the Reporting of Observational Studies in Epidemiology (STROBE) Statement [8].

1. Cohort:

The data of patients, all suffering from PAD with an infragenicular pathology and treated between March 2021 and February 2022, were retrospectively analyzed. Inclusion criteria for this study were BTKA application and completed ankle-brachial index (ABI) measurement pre–intervention, as well as a post-intervention duration of 2 weeks and 6 months, respectively. 

The vascular statuses in all patients were determined via ABI and Doppler pressure, defined according to global vascular guidelines on the management of chronic limb-threatening ischemia [9], and the target vessels and lesion length were measured via angiography. To further classify the extent of stenosis, a runoff-score was measured [10]. The ABI was calculated via the highest vessel pressure in the lower extremity. Every patient received a 2 week and 6 month follow-up including ABI measurement and PAD stage reassessment compared to their pre-interventional status. Additionally, every patient with PAD stage 5 received a photo-documentation of their wound/ulcera status, which was described and documented according to the WIfI classification [11]. Before the intervention, patients presented with PAD stages between 2 and 5. Two of the patients had a prior open vascular surgery on the contralateral side of the vessel targeted in this procedure, and all others had no history of open vascular surgery.

2. Atherectomy Device:

The Phoenix rotational atherectomy ™system (RAT), 1.5MM X 149CM, 4F, (Philips, San Diego, CA, USA), is a flexible double-lumen catheter able to cut out and capture arterial plaques, soft and calcific, with a front cutter. The device used on all patients from this study had a diameter of 1.5 mm. It contains a torque shaft and an attached cutter to remove the plaques intraluminally [12,13].

3. Procedure:

The procedure was initiated by cannulating the ipsilateral femoral artery and inserting the atherectomy device via a sheath while both identifying the lesion and controlling success via angiography. A total of 12 out of 33 (42.42%) patients received additional angioplasty via drug-eluting balloons. No stenting procedures were performed. The mean procedure time was 64 min, varying from 31 to 147 min. After the procedure, an angioseal system was used to prevent bleeding incidences. The mean length of the hospital stay was 4 days.

**Figure 1:**
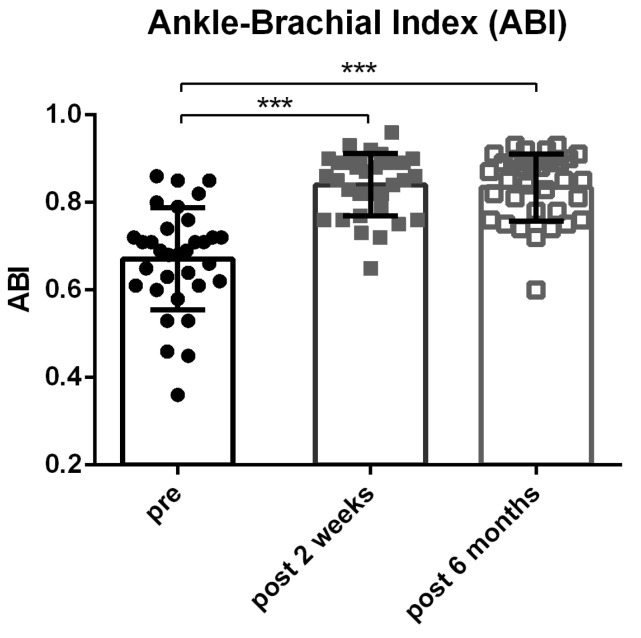
Ankle-Brachial-Index at pre-intervention and at time marks of 2 weeks and 6 months. ABIs were acquired according to ESVS Guidelines (mean ± SD). (*** = *p* < 0.001).

4. Statistical analysis:

Data analysis was performed using GraphPad Prism 6 (GraphPad, La Jolla, CA, USA) and SPSS 26 (Statistical Package for the Social Sciences, Inc., Chicago, IL, USA). All data are shown as mean ± SD for *n* = 33 patients. For the data analysis, a Friedman test for repeated measurements (Figure 1) was used, followed by a Dunn’s test for comparison between groups. Analysis differences were assumed to be significant with *p* < 0.05.

## 3. Results

After the application of inclusion criteria, 33 patients with a mean age of 72.46 ± 11.85 (SD) were included. A total of 13 out of 33 patients were female (39.4%), and 46% of patients were current smokers with mean pack years of 12.33 ± 11.99 (SD). Most patients presented with other preexisting comorbidities, e.g., 66.7% with arterial hypertension or diabetes mellitus. For more details, see Table 1. 

About half of the interventions were performed on the anterior tibial artery (16 out of 33), while 12 were performed on the posterior tibial artery and 5 on the fibular artery (Table 2). The run-off score was determined with a mean of 7.44 ± 2.17 (SD).

Among all the interventions, one complication occurred: An acute bleeding at the puncture site made an emergency surgery necessary, and a bovine patch was attached to the bleeding vessel. No additional intervention was needed. No vessel dissections were observed, and no limb amputations during the follow-ups were necessary.

In the two-week checkup, the mean ABI significantly increased from 0.67 ± 0.12 (SD) to 0.84 ± 0.07 (SD) (*p* < 0.0001). After six months post-intervention, no further change could be observed (mean 0.83 ± 0.08 (SD)) (*p* < 0.0001 compared to baseline) (Figure 1). 

At 6 months post-intervention, the number of patients in PAD stage 5 decreased by 15.2%. At 6 months post-intervention, 18.2% of all patients were at PAD stage 5 (Table 3) according to the clinical examination.

## 4. Discussion

Atherectomy via the Phoenix atherectomy ™system can improve the PAD stage in patients. In stage 5 in particular, with chronic wounds, the risk of infection and major amputation is high and related to an increased mortality rate [14]. In this retrospective study, the proportion of stage 5 PAD could be reduced by a third by the usage of atherectomy in the BTK area. This novel area of application has not been described in the literature so far, as relevant and justifiable doubts exist regarding atherectomy in small and calcified vessels and as experiences are rather limited and so far only exist for critical limb ischemia [15,16]. However, in our retrospective study, ulcera healed completely during the six months in a relevant portion of patients. In contrast to our findings, a recently published meta-analysis showed a very modest improvement in ABI’s and patency rates. This difference could be explained by the high heterogeneity regarding the patient cohorts included in this study or the difference regarding the intervention technique. Additionally, the number of stage IIa patients went from nonexistent to 18.2%. This leads to the conclusion that after six months, a significant part of the cohort reached a stage regarding their PAD where additional surgical interventions were no longer necessary [17,18]. Comparing the two-week and six-month follow-ups, a positive trend regarding clinical symptoms becomes apparent, while the mean ABI values remain constant. This finding may emphasize the importance of the clinical symptoms as outcome parameters instead of purely focusing on patency rates when evaluating a successful treatment, which can be found in comparable studies [19,20]. With just one short-term complication and no long-term complications, the intervention seems feasible to accomplish in comparison to similar procedures, when routinely performed by a specialized team [21]. The observed complication was on the puncture side. Complications like an embolism or dissection did not occur.

Even if these findings look promising, relevant limitations have to be considered while assessing the impact of BTK atherectomy for PAD patients. This study is a retrospective data analysis from a routine intervention with a wide range of preexisting conditions, PAD stages and age differences (Table 1 and Table 3). Since the patients were treated in a multiprofessional context together with physical therapists, general practitioners, diabetologists, etc., there are different impact factors regarding the post-interventional outcome. On the other hand, our cohort represents a real-life variety of patients with symptomatic PAD. Despite the heterogeneity of the group, there was a clear narrowing of the SD among the ABIs post-intervention, which can be interpreted as a direct effect of the intervention itself.

This study is a first assessment of the feasibility of the Phoenix™ 1.5 mm atherectomy system, and a long-term reassessment seems to be urgently required to evaluate the impact of this novel device. This study cannot include information about long-term vascularization after intervention. Still, the ongoing wound healing process in the PAD stage 5 patients is very promising. Our findings are not similar to those published in the already mentioned meta-analysis. Here, a less favorable improvement of wound healing was observed [22]. One explanation could reside in the difference regarding the performed therapy; furthermore, it could underline the advantages of atherectomy over sole angioplasty in the BTK area, even though the literature shows no difference in the mortality and amputation rates [23]. This study was conducted to assess the feasibility and patency of BTKA with the Phoenix™ catheter in one center as a pilot study. This study adds to already existing and promising outcomes of atherectomy systems in the above-the-knee arteries and could become an additional method for improving long-term patency [24]. The impact of the high expertise of the performing interventionalist must be mentioned, while a relevant risk of a confounder as well as a lack of transferability may exist. Further studies should address the effectiveness of revascularization therapy compared to conservative treatment according to the current ESVS guidelines [9]. However, this study supports the existing data on interventional success and safety in BTKA [20]. 

Furthermore, not only did the mean ABIs increase significantly, but there was also a remarkable shift toward lower PAD stages. In accordance with this, in this study, the most relevant clinical improvements could be observed in stage 5 PAD patients. Although ABI and PAD stages are routinely used assessment tools for clinicians, other tools such as transcutaneous oxygen pressure or near-infrared spectroscopy could also be helpful to gather more information to evaluate patient outcomes and therapeutic success [25]. These could strengthen the described findings in future studies.

## 5. Conclusions

Phoenix™ atherectomy usage in the BTK area seems to be feasible and related to a favorable outcome in this retrospective study, and a significant increase regarding the mean ABI measurement was observable.

## Figures and Tables

**Table 1:** Preexisting conditions.

Characteristic	Value
Gender	
Male	20 (60.6)
Female	13 (39.4)
Active smokers	15 (45.5)
Pack years, mean (range)	12.3 (1–38)
Diabetes mellitus	22 (66.7)
Hypercholesterolemia	18 (54.5)
Arterial hypertension	22 (66.7)
Coronary heart disease	7 (21.2)
Obesity	4 (12.1)
Chronic kidney disease	11 (33.33)
Prior endovascular interventions	12 (36.36)
Prior open vascular surgery on ipsilateral side	0 (0.0)

Values are a number (percentage) unless stated otherwise.

**Table 2:** Target vessel for atherectomy.

	Fibular Artery	Anterior Tibial Artery	PosteriorTibial Artery
Target vessel, *n* (%)	5 (15.15)	16 (48.48)	12 (36.36)

**Table 3:** Peripheral arterial disease (PAD) stages; PAD classification according to Rutherford pre- and post-intervention.

PAD Stage *n* (%)	Pre-Intervention	Post-Intervention 2 Weeks	Post-Intervention 6 Months
1		12.1 (4)	18.2 (6)
2/3	27.3 (9)	30.3 (10)	24.2 (8)
4	27.3 (9)	15.2 (5)	12.1 (4)
5	45.5 (15)	42.4 (14)	30.3 (10)

## Data Availability

The datasets generated during and/or analyzed during the current study are available from the corresponding author on reasonable request.
